# The Effect of Binder Loading on the Pore Size of 3D Printed PMMA

**DOI:** 10.3390/ma14051190

**Published:** 2021-03-03

**Authors:** Simon Riechmann, Odo Wunnicke, Arno Kwade

**Affiliations:** 1 Institut für Partikeltechnik, TU Braunschweig, Volkmaroder Str. 5, 38104 Braunschweig, Germany; a.kwade@tu-braunschweig.de; 2 Evonik Operations GmbH, Creavis, Paul-Baumann-Str. 1, 45764 Marl, Germany; odo.wunnicke@evonik.com

**Keywords:** binder jetting, porosity, tensile strength, 3D printing, PMMA

## Abstract

Binder jetting is known to produce porous objects by depositing the binder selectively layer by layer on a powder bed. In this study, the pore size of printed parts and the correlating mechanical properties are investigated on a commercially available PMMA powder binder system. Pore sizes are measured via capillary flow porometry and mechanical properties via tensile tests. Porometry indicates that the pore size stays at a constant level of 22 µm at 5 to 10 wt% binder loading before decreasing to 6 µm at loadings of 30 wt% or higher. The results were compared with the mechanical testing and related to the agglomerate strength model of Rumpf. The highlights of the article are the application of a binder jetted part as a filter and the identification of a close relationship between porosity and mechanical strength, similar to phenomena in agglomeration science.

## 1. Introduction

Binder jetting (BJ), originally called 3D printing, was developed and patented at the Massachusetts Institute of Technology in 1993 [1]. A binder is selectively deposited via inkjet printing onto a powder bed. The powder bed is then lowered, and the next powder layer is coated on top of the powder bed. One advantage of this technology is that any powder material or even multiple powders can be applied [2]. Other advantages, compared to other 3D printing technologies, include the large build size, easy scalability, and high speed of the process [3]. A disadvantage of binder jetting is that the parts are highly porous after printing and have limited mechanical strength. Typically, such parts are further processed, e.g., infiltrated with an epoxy resin [2]. A good overview over the state of the art can be found in the review form Mostafaei et al. [4]. The porosity of Binder jetted parts can also be benfecial for some applications. For instance, He et al. [5] and Hwa et al. [6] printed clay ceramics for water treatment and could reduce the turbidity using a 3D printed filter. Lee et al. [7], Low et al. [8], and Tijing et al. [9] reviewed the possibilities to use 3D printing in membrane technology. In the work of Goole and Amighi [10], several powder binder combinations for printing pills are described. Brunello et al. [11] reviewed the research on powder-based 3D printing technologies for tissue engineering. More recently, Ke and Bose [12] printed scaffolds out of tricalcium phosphate and investigated the influence of the morphology on the physical, mechanical, and biological properties. Yu et al. [13] showed the possibility of printing tablets with a material gradient, and therefore could control the release retardation behavior. Several authors have shown the feasibility of such systems, but further research to provide a fundamental understanding how process and material interact with each other is needed. To do so, it is necessary, to understand the relationship between porosity and mechanical properties. Some authors have investigated porosity, e.g., Spath et al. [14] printed 3D scaffolds from hydroxyapatite. As a binder, an aqueous solution of dextrin and saccharose was used. In their study, the particles are fractioned via a sieve shaker into different particles sizes and later recombined to print bimodal powder mixtures. Due to better packing, the printjob using a bimodal granule blend has a higher mechanical strength (~16 MPa) and porosity ϵ (ϵ = 54%) compared to the printjobs using individual fractions (10 MPa and ϵ = 52%). Polzin et al. [15] were able to create porous ceramics from alumina and silicon carbide with up to 69.6% porosity and mention a potential application in filtration applications. Maleksaeedi et al. [16] added an infiltration process after the 3D printing of alumina to reduce porosity and pore sizes. The pore sizes show a bimodal distribution with a maximum at 0.1 µm pores and a second maximum between 10 and 100 µm pores. With the infiltration the mechanical properties improved up to 15 times compared to the non-filtrated part. Zocca et al. [17] printed SiOC ceramics out of preceramic polymers with 20% porosity. Yet, a model to describe the relationship between porosity and tensile strength does not exist. In this study, the focus is on measuring how the binder loading influences the porosity and the pore size distribution and connecting this information to the mechanical properties by applying the knowledge of the agglomeration science developed by Rumpf [18] and Schubert [19,20], and fitting their model to the obtained experimental results. This knowledge then can be applied for specific applications and consequently for controlling the porosity of binder jetted parts. A good understanding of controlling the pore sizes can push forward new applications for binder jetting technology which are currently under research, namely, membranes or filters with controlled porosity, printing pills with adjusted dissolution behavior, scaffolds for tissue engineering or gradient materials. As a variable, the binder loading was varied to investigate the effect on the form fidelity, porosity and mechanical properties. Furthermore, we transferred the gained knowledge into a filter application and showed its feasibility.

## 2. Materials and Methods

As a powder binder system, a PMMA powder with a median particle size of d_50,3_ = 55 µm and PolyPor C as a binder were used. The materials were supplied by Voxeljet AG, Friedberg, Germany. PolyPor C is a solvent mixture of different dibasic esters. The powder and binder were processed as received. The 3D printer, VX200 from Voxeljet AG (Friedberg, Germany), equipped with a Fujifilm Spectra Nova JA 256/80 AAA printhead (Fujifilm, Tokyo, Japan) was used to print samples at binder loadings between 5% and 45%.

The binder loading (BL) is calculated by the quotient of the mass of a single droplet (m_droplet_) and the total mass per Voxel using following equation [21]:

(1) $$\mathrm{BL}=\frac{m_{\mathrm{droplet}}}{m_{\mathrm{droplet}}{\;+\;m}_{\mathrm{powder}}}\cdot 100\%$$

In Equation (1), m_droplet_ is measured by printing and weighing 10,000 droplets from each nozzle and dividing it by the total number of droplets. This is done before and after the print job to assure stable printing conditions. The droplet mass m_droplet_ is highly dependent on the frequency. Frequency and resolution in printing direction (dx) define the speed of the printjob. Therefore, smaller voxel sizes require higher frequencies to have similar process times. The mass of the powder m_powder_ is calculated from the voxel volume V_voxel_ and the bulk density ρ_Bulk_:

(2) $$V_{\mathrm{voxel}}\;=\;\mathrm{dx}\cdot \mathrm{dy}\cdot \mathrm{dz}$$

(3) $$m_{\mathrm{powder}}{\;=\;V}_{\mathrm{Voxel}}\cdot \rho_{\mathrm{Bulk}}$$

In Equation (2), dx and dy are the printing resolution, defined in the printer settings and dz the layer thickness. The bulk density in Equation (3) was 725 kg/m^3^ according to the supplier Voxeljet AG. Therefore, by decreasing the voxsize by adjusting the print resolution, a higher BL can be realized.

**Figure 1:**
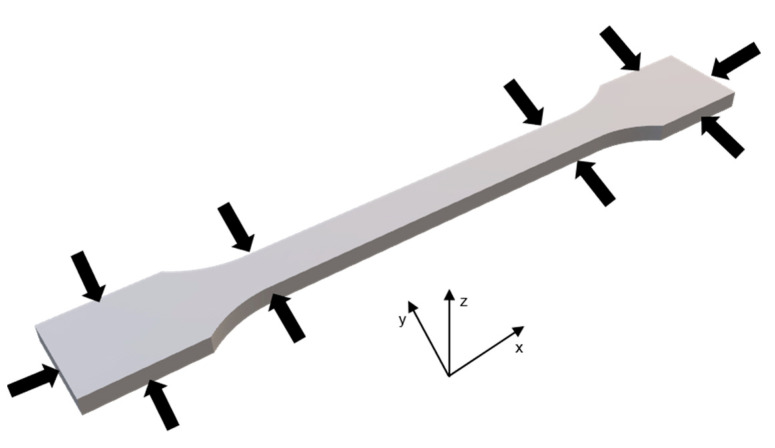
Print orientation of tensile roads in powder bed and measurement points for shape deviation analysis.

The stl-files of the CAD parts were sliced using RapiX 3D (FORWISS, University Passau, Passau, Germany) and printed with a layer thickness of dz = 150 µm. The orientation in the printer is depicted in Figure 1. In Table 1, the different print settings are shown. The binder loadings before and after printing, calculated over all the printjobs, have a standard deviation of 0.52%, which results in different values for the BL in the print job, compared to the target BL. Therefore, the calculated binder loadings are also shown in Table 1. For easier comprehension, in the results and discussion only the target binder loadings are applied. To provide a uniform powder spread, 2 mm of powder are coated before the start of a printjob.

For post processing, parts were kept in the powder bed for 24 h allowing for a diffusion of the solvent, as recommended by Voxeljet AG. Afterwards the samples were removed from the powder bed and kept for at least 42 h at room temperature to let the solvent partially evaporate. To remove residual solvent, a post processing step was applied, and the samples were placed in an oven for 5 h at 70 °C. Within each print job, 4 tensile rods (DIN EN ISO 527 [22] Type 1A) and multiple spherical chips with a diameter of 12 mm and a printing thickness of 1.5 and 2.1 mm for pore size and porosity evaluation were printed.

The shape deviation (SD) was determined after post processing by measuring the width and height at four different points (see Figure 1) and the length of the tensile rod.

From the measurements, two different values were calculated: the in-plane variation describing binder migration into the sides (SD_x-y_), only taking the length and width of the sample into account, and the height deviation describing binder migration into the bottom of the printed part (SD_z_). To calculate the SD, the following equation was used:

(4) $${\bar{\mathrm{SD}}}_{i}\;=\;\frac{1}{n}\cdot \overset{n}{\underset{k=1}{\sum }}\left(\frac{X_{\mathrm{measured},i}}{X_{\mathrm{model},i}}\;-\;1\right)\cdot 100\%$$

The index i represents for x-y or z direction, n is the number of measurements for the according length measurement (e.g., 16 for the height measured on 4 positions on 4 tensile rods), X_measured_ is the measured length value, and X_model_ is the corresponding original size of the CAD model.

The tensile strength and elongation at break were measured using a Zwick Roell Retro Line. The measurements are following DIN EN ISO 527-1 [22] with an initial load of 10 N and a speed of 50 mm/min. For each print setting four tensile rods were tested, and the mean and standard deviation were determined.

Porosity was measured indirectly by the determination of the density of the parts following ASTM B962 [23] using the Archimedes principle. For this measurement, small spherical printlets with a diameter of 12 mm and a thickness of 2.1 and 1.5 mm were used. In a first step the mass of the sample was determined (m_A_). Afterwards the samples were immersed in oil, removed from the oil and the surface oil was cleaned off. The purpose of this treatment is to fill the porous volume. The mass of the impregnated printlet is measured (m_B_). In the third step, the mass of the oil-impregnated samples in water (m_C_) was determined. From the three masses and the density of water ${(\rho }_{w})$, the part density can be determined following the equation:

(5) $$\rho_{\mathrm{print}}\;=\;\frac{m_{A}\cdot \rho_{w}}{m_{B}{\;-\;m}_{C}}$$

More details can be found in the ASTM B962 [23]. Based on the density of the printed part and the material density of PMMA ($\rho_{\mathrm{material}}$ = 1190 kg/m^3^ [24]), the porosity (ϵ) was calculated using the equation:

(6) $$\epsilon \;=\;\left(\;1\;-\frac{\rho_{\mathrm{print}}}{\rho_{\mathrm{material}}}\right)\cdot 100\%$$

The pore size distribution was determined via capillary flow porometry (Porolux 1000^TM^, Belgium). The Porolux measures the flow of nitrogen against the applied pressure. For the printed samples, a pressure of 0.2 bar was sufficient to remove all the wetting liquid from the sample. With this pressure, pores up to 3.2 µm can be measured following the Young–Laplace Equation. A more detailed description of the method is found in the research from Jena et al. [25].

For this purpose, the printlets with 12 mm diameter and 1.5 mm nominal thickness were used. Before measuring, the samples were immersed into Porofil liquid (γ = 16 mN/m) and placed under vacuum (<100 mbar) to wet all pores inside the body.

For SEM micrographs, a Hitachi TM 4000 Plus was used (Hitachi, Tokyo, Japan), and samples were sputtered with 10 nm of gold using a Quorum Q150R Plus (Laughton, United Kingdom). The samples for SEM micrographs were obtained from the tensile test and cut to fit the SEM. The investigated surface on the micrograph is the fracture surface after the tensile testing.

**Figure 2:**
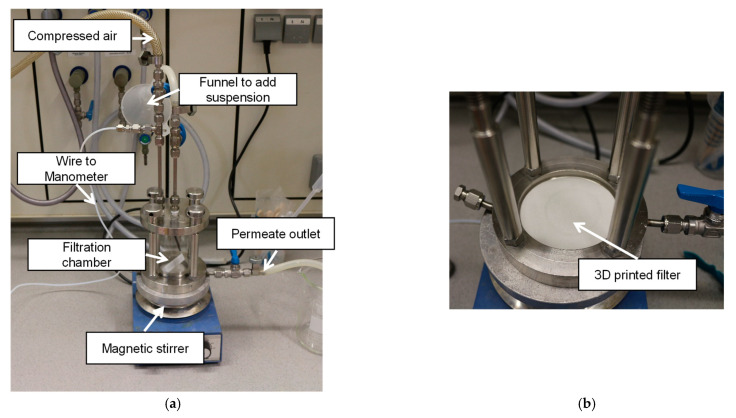
Self-build filtration set-up, for pressures up to 0.5 bar. (**a**) Set up with external utilities, (**b**) image of 3D printed filter placed inside the setup.

For the filtration experiments, a self-built set-up was used that can be seen in Figure 2. With this set-up, a dead-end filtration using compressed air (up to 0.5 bar) was executed. The compressed air presses the liquid and small particles through the printed membrane and the bigger particles are retained. As feed, a suspension of Vestosint (Evonik Resource Efficiency GmbH, Essen, Germany) particles in water was used. To avoid clogging, the liquid was stirred with a magnetic stirrer, without being in direct contact with the membrane (see Figure 2a).

In order to monitor the filtration process, the particle size in the feed suspension and the permeate were measured. For measuring the particle size laser diffraction was applied (Mastersizer 2000, Malvern, UK) with a Hydro 2000S unit for liquid samples. Before measurement, the samples received 1 min ultrasonication to induce deagglomeration. The measurement results in a volume-based particle size distribution, therefore particle sizes will be marked with the number 3 as index.

## 3. Results

### 3.1. Shape Deviation

**Figure 3:**
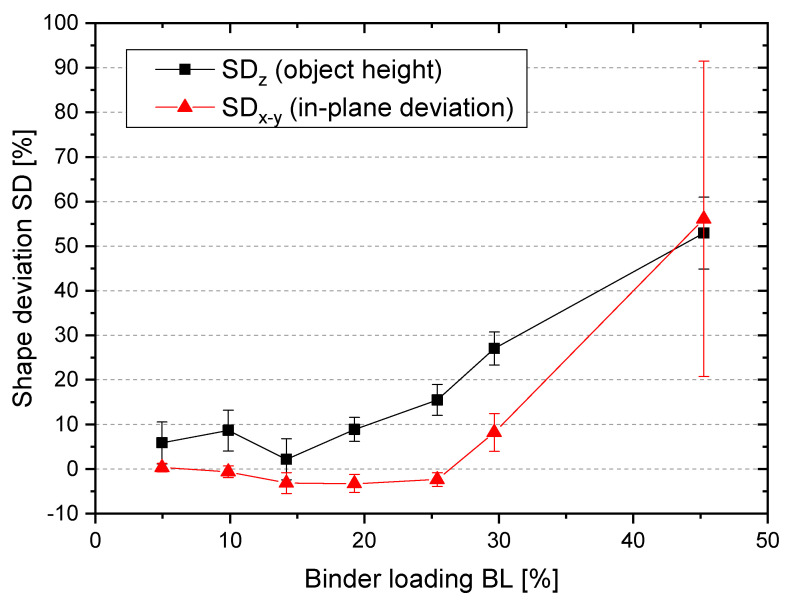
Shape deviation at different binder loadings.

In layer-wise 3D printing technologies such as binder jetting and selective laser sintering (SLS), shape deviations often occur [26,27,28]. In the following, the 3D printed tensile rods at different binder loadings were measured and the SD was calculated according to Equation (4). The dependency of shape deviation as function of the BL is shown in Figure 3. The error bars in Figure 3 display the standard deviation from four measured samples. Different effects are observed for the deviation in-plane compared to z-direction. The in-plane deviation is very small (<0.6%) for BL ≤10%. For BL up to 25%, a small shrinkage (2.3–3.2%) of the parts is measured. For loadings of 30%, the SD_x-y_ increases to 8.2%, and at 45% the SD_x-y_ is 56.1% (see Figure 3).

**Figure 4:**
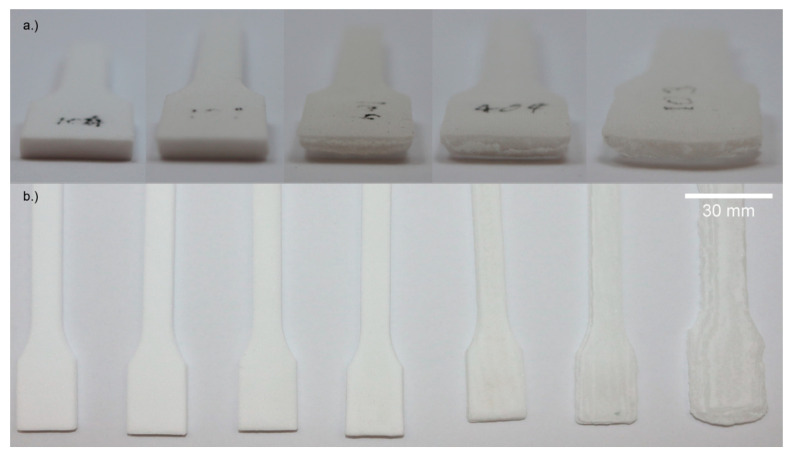
Optical micrographs of tensile test specimen. (**a**) Front perspective, from left to right with 5, 20, 25, 30, and 45% BL; (**b**) top perspective, from left to right with 5, 10, 15, 20, 25, 30, and 45% BL.

In the z-direction, the parts are always larger compared to the model shape. For BL ≤ 20%, the SD_z_ is still moderate (<9%). For BL of 25% and higher, the SD_z_ increases strongly (up to 52.9% at 45% BL). For BL from 30% upwards, a trapezoid shape can be observed (see Figure 4a,b), and for 45% a complete loss of shape accuracy. The inhomogeneous shape at a BL of 45% leads to the highest standard deviation for the in-plane shape deviation SD_x-y_. In Figure 4a, the different thicknesses are shown. Figure 4b depicts that up to 15% BL the shape is accurate, at 20% BL the shape, especially in the necking area of the tensile rod, is smaller compared to smaller loadings. At higher loadings, the width of the samples increases, and the surface is roughened.

### 3.2. Mechanical Properties

**Figure 5:**
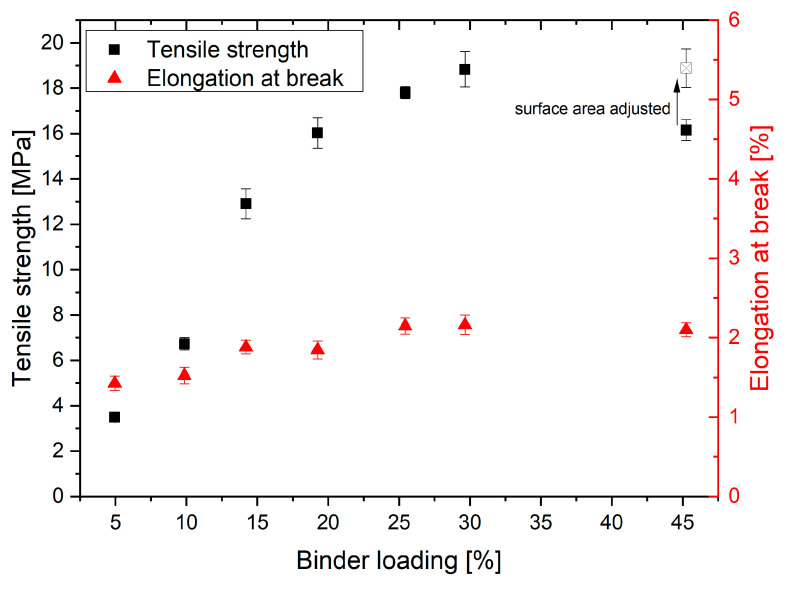
Tensile strength of samples at different binder loadings (BL). Due to the heavy trapezoid shape at 45% BL, the tensile strength was adjusted by determining the actual surface area via optical microscope.

The PolyPor C system is based on a solvent bonding mechanism. The printed solvent dissolves particles partially and fuses adjacent particles together. In the following section, this effect is analyzed, and the influence of the BL on the mechanical properties is investigated. The tensile strength (TS) and elongation at break in relation to the BL is depicted in Figure 5. For BL ≤30%, a strong increase from 3 MPa at 5% BL to 18 MPa at 30% BL is observed. At 45% BL, the tensile strength decreases slightly to 16 MPa. The elongation at break increases gradually from 1.4 to 2.1% for the increase in BL from 5% to 30%.

**Figure 6:**
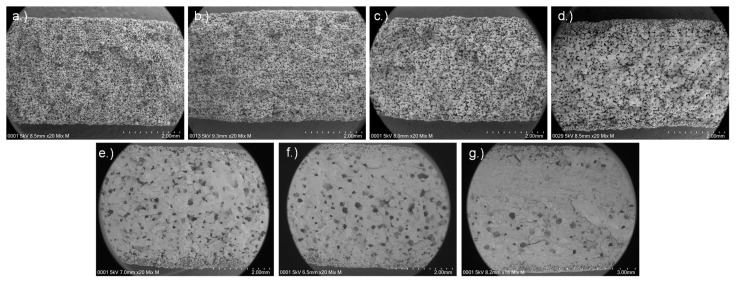
SEM micrographs at 20× magnification from the broken edge of tensile test specimen with different binder loadings: (**a**) 5 wt%; (**b**) 10 wt%; (**c**) 15 wt%; (**d**) 20 wt%; (**e**) 25 wt%; (**f**) 30 wt%; (**g**) 45 wt%.

**Figure 7:**
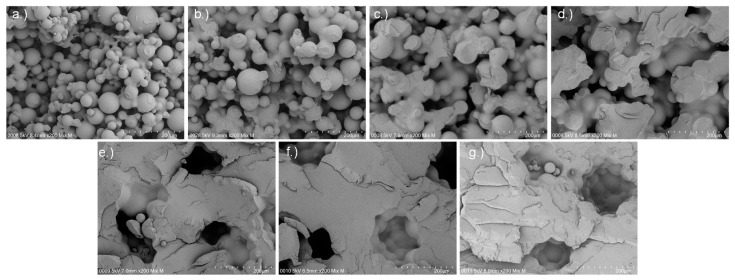
SEM micrographs at 200× magnification from the broken edge of tensile test specimen with different binder loadings: (**a**) 5 wt%; (**b**) 10 wt%; (**c**) 15 wt%; (**d**) 20 wt%; (**e**) 25 wt%; (**f**) 30 wt%; (**g**) 45 wt%.

The increase in tensile strength is explained by the higher saturation at higher BL. Due to the higher saturation, more of the particles dissolve, and therefore stronger connections between the particles are formed. Low loadings result in only small and narrow bridges between the particles. At higher loadings (≥25% BL), the particulate structure is lost, i.e., almost all particles are dissolved by the binder, and a network with larger pores is formed. The SEM micrographs in Figure 6 and Figure 7 illustrate this effect: at 5% BL, only very narrow bridges between particles are observed. These bridges enlarge when increasing the loading up to 20% BL. At 25% BL, particles are only present at surface of the sample, while the center is rather dense with coarser pores inside. The decrease in tensile strength at 45% BL is caused by the high shape deviation. During tensile testing, the width and height of the specimen is measured at the widest part. After testing, the actual surface area is measured with an optical microscope, and a corrected tensile strength is calculated (see Supplementary Information Figure S1). The corrected value is 18 MPa and similar to the 30% BL specimen. This leads to the conclusion that at 30% BL the maximum of tensile strength is reached, and a further increase in BL does not change the TS. In contrast, optimum BL is between 20 and 30%, depending on whether mechanical properties or shape accuracy is in focus of the application.

The coarse pores are most likely formed during the post processing. During the drying process the solvent is evaporated from the outside layers and consequently a denser and harder to permeate layer for the remaining solvent is formed, which in the end leads to blister formation. This process in known from the lacquer industry [29].

### 3.3. Porosity and Pore Size Distribution

**Figure 8:**
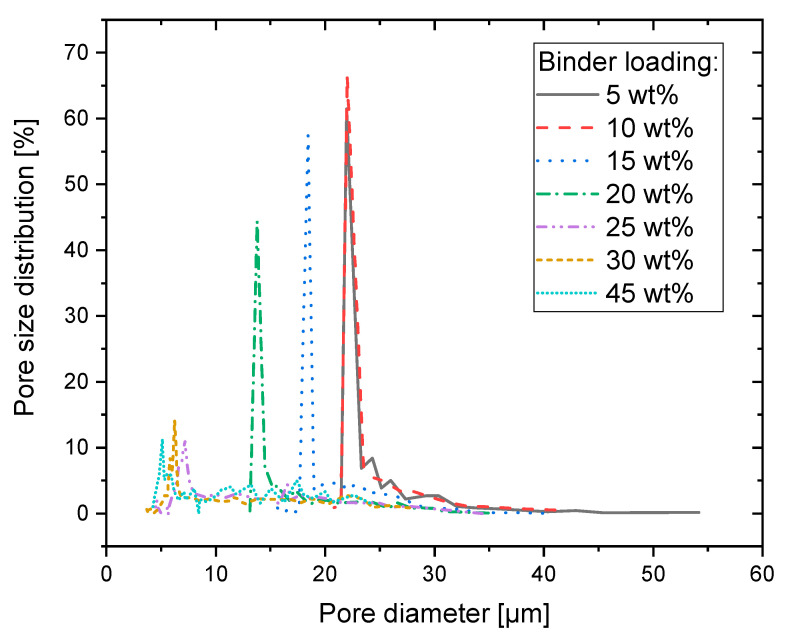
Pore size distribution obtained by capillary flow porometry. Pore sizes decrease with increasing BL from 23 µm to 6 µm.

The pore size distribution of different BLs is measured using capillary flow porometry. Figure 8 shows the pore size distributions at different BLs. For 5% to 10% BL, the pore sizes are almost identical. For BL > 10%, increasing binder loading results in a decrease in the pore sizes. In general, all the pore size distributions show a distinct peak at their smallest pore size. For BL up to 20%, the peaks account for more than 40% of pores, thus by adjusting the binder loading control of the distinct main pore size is possible. In contrast, at BL of 25% and higher, only 10–12% clearly accounted for one size, and the rest of the pores are distributed over a wide range of sizes.

**Figure 9:**
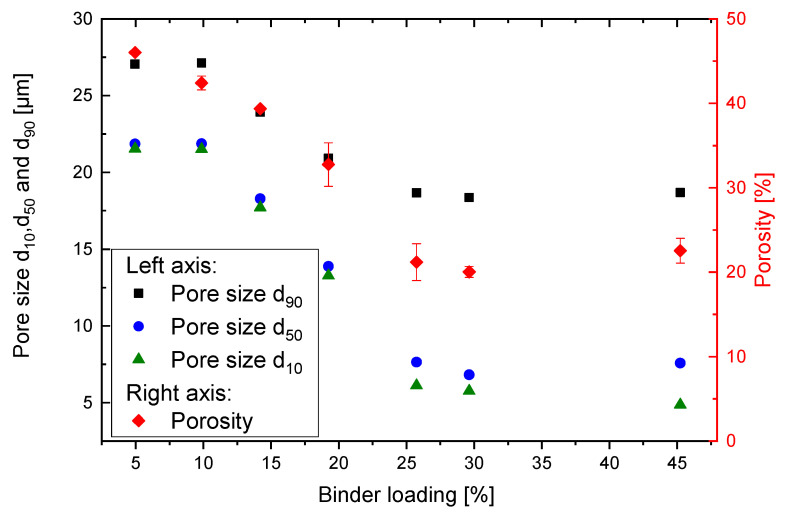
Porosity and pore size d90, d50, and d10 at different binder loadings. The pore sizes and porosity follow a similar trend. After a decrease between 20% and 25% BL, the values reach a constant level.

For a more distinct comparison and further analysis of the pore size distribution, the 10th (d_10_), 50th (d_50_), and 90th (d_90_) percentile of pores sizes are calculated and shown in Figure 9. In addition to pore size distribution, also the porosity itself is crucial and obtained by ASTM B962 measurements using the Archimedes principle. The results are depicted in Figure 9.

In general, even with very high binder loadings the particles cannot be completely fused together and a porosity of 20–25% with pore sizes in the micrometer range still exists. This is expected as the printed solvent is mostly evaporated in the post processing, and therefore pores must be formed. Nevertheless, it is shown that with printing process itself, the microstructure and with it the median pore size can be controlled in a range of between about 22 µm and 6 µm. With the latter, filter applications or tissue engineering are already well feasible.

### 3.4. Application of Printed Structures as Filter

The obtained results from pore size analysis and mechanical properties were promising, thus an actual filtration test was performed. For this test, a specimen with a 2 mm thickness and a diameter of 75 mm was printed with the same setting as the 30% BL samples. Applying an excess pressure of 0.5 bar of compressed air for 3 minutes, the liquid and small particles are pressed through the 3D printed specimen while larger particles are retained.

**Figure 10:**
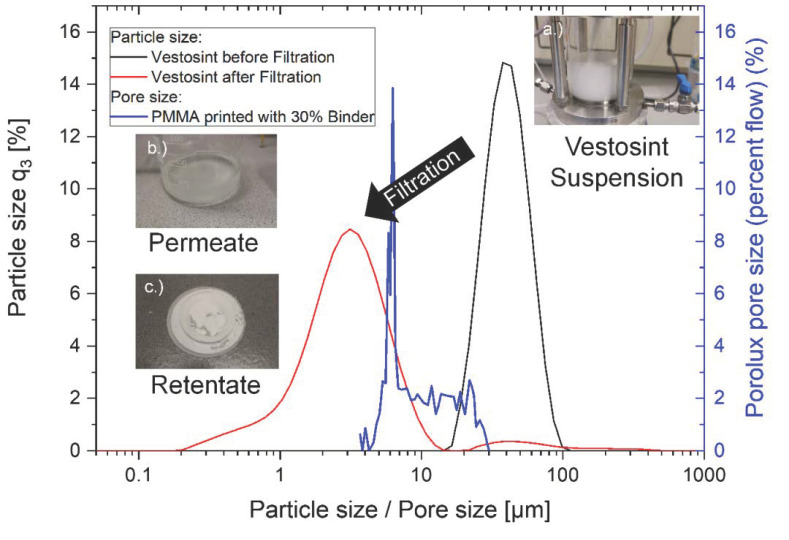
Pore size of printed specimen used in filtration device (blue) in comparison to the particle sizes before (black) and after (red) filtration. Inlet images: (**a**) turbid suspension (feed); (**b**) picture of permeate; (**c**) picture of filter cake on printed filter.

In Figure 10, the volume-based particle size distribution and pore size distribution for a filter experiment with a 3D printed filter are shown. The particle size decreases from a d_50,3_ ~ 50 µm to a d_50,3_ ~ 4.5 µm after the filtration. This correlates well with the median membrane diameter of d_50,pore_ = 6.4 µm. Due to the measurement of a volume-based particle size distribution, the small Vestosint particles are suppressed in the measurement and not shown in the feed suspension. Only after filtration, when the concentration of larger particles is reduced, can the smaller particles be measured by laser diffraction. The inlet image in Figure 10a depicts a turbid suspension of the Vestosint particles in water. After this, the filtration a permeate (see Figure 10b) and retentate (see Figure 10c) are formed. It can be observed that the turbidity of the permeate is highly reduced compared to the feed suspension. The reduced turbidity is a sign for lower concentration and smaller particles compared to the feed suspension. Further, a filter cake is formed on top of the printed filter.

The preliminary filtration results show that with a change in the BL, the pore size can be adjusted, and this adjustment can be used for actuals separation tasks.

## 4. Discussion

The shape deviation in the presented study is caused mainly due to binder migration. To ensure homogenous printing, 2 mm of powder was coated under the printed parts. At higher loadings, the binder diffuses into these lower layers and a leaking effect occurs. Regarding the in-plane deviation for BL ≤ 10%, the binder is accurately placed by the inkjet process, and therefore almost no deviation occurs. When increasing the loading, more of the powder is dissolved, and the part shrinks slightly (≤25% BL). At higher loadings, the trend is similar for the z-direction, as well as for the in-plane measurements. This is because at these loadings the powder bed cannot take in all the binder, and a migration into the surrounding powder bed takes place. Additionally, the trapezoid shape can be explained by this. For the first few layers, the binder migrates mainly into bottom layers. As soon as they are filled and cannot take in more binder, the binder starts to migrate into the sides.

Kellner [26] and Schmutzler et al. [27,28] also investigated the shape deviation, particularly the trapezoid shape and curling of the PMMA-based 3D printed system. Instead of using a solvent based system, they used a reactive system and explained the deformation by an inhomogeneous shrinking.

For the system applied in this work, this explanation is not applicable because as seen in Figure 3 with higher loadings, our parts lose the shape and become larger than the model size. Therefore, in our case the deformation is due to the excess binder and its migration into the surrounding particle bed.

The tensile strength of casted PMMA is up to 80 MPa, and an elongation at break of up to 5.5% [24]. Due to the nature of the process, a decline in performance is expected. Other authors investigated similar systems: Polzin et al. [30] for example investigated a blend of PMMA and polyethylmethacrylate and achieved a tensile strength of 2.91 MPa with an elongation of 1.4%. In the publication, only the droplet mass and printing resolution are given, but not the binder loading, thus a direct comparison is difficult. Patirupanusara et al. [31] blended PMMA with maltodextrin and polyvinylalcohol. They could achieve a flexural strength of ~1 MPa with the highest binder loading. Therefore, the achieved strength of 18 MPa is a very good value for binder jetting. However, it is noteworthy that there is a tradeoff between mechanical properties and shape accuracy. The optimal printing conditions are 20–30% BL, depending on the application. For smaller pores, higher loadings are required, and for a better shape accuracy the lower loadings are favorable.

The porosity of the printed system between 40% and 50% at a BL of 5–10% (see Figure 9) is similar to what other authors could achieve with binder jetting technology. Spath et al. [14] printed scaffolds from hydroxyapatite and could achieve a porosity of 54% and a mechanical strength of 16 MPa. Polzin et al. [15] made porous ceramics form alumina and silicon carbide with a porosity of 69.6%. The porosity of 23% at a BL of 30% is therefore a remarkable result. Zocca et al. [17] could achieve a similar porosity with the use of preceramic polymers to print a SiOC ceramic. Nonetheless, most of the research regarding binder jetting and porous parts deals with ceramic parts, which always need to undergo an energy consuming sintering process step. In contrast to the previously mentioned literature, we try to directly obtain a microstructure applicable for filtration.

Pore size distribution of binder jetted parts are at this point not known to the authors. The distribution of the pores sizes is a field with a high potential for future studies. The insight about the microstructure can help in understanding the process better and designing new applications.

Closely observing the mechanical data and the porosity, a similar trend is observed. While mechanical properties increase with an increase in BL, the porosity decreases in a similar fashion. In the following paragraph, we aim to connect the results obtained with the fundamental research for agglomerate strength and wetting from Rumpf [18] and Schubert [32] to the obtained measurement results. In general, the wetting of a powder bed can be classified into three regimes [33] depending on the saturation.

For saturations up to 30% [20], single bridges are formed, and the agglomerate strength can be described by Equation (7) [18]:

(7) $$\sigma_{z}=\frac{1\;-\;\epsilon }{\epsilon }\cdot \frac{H}{x^{2}}$$

where σ_Z_ is the maximum transferable tensile strength, ϵ the porosity, H the adhesion force of the bonding bridge, and x the particle size. In the derivation of this equation several assumptions are made [34]:

- The particles are convex and randomly distributed;
- The adhesion force distribution is the same over all contact points;
- The mean number of neighbors k, where adhesion forces are present, can be described by k ≈ π/ϵ

.

For very high saturations (<80% [34]), the powder is filled with liquid and the maximum tensile strength of wet agglomerates is given by Equation (8) [33].

(8) $$\sigma_{z}\;=\;S\cdot p_{k}$$

In Equation (8), S is the saturation and p_k_ the capillary pressure. This equation is used to describe the saturation regime. In between the bridge and saturation regime there is a transition regime. In this regime, both parts are partially present, and therefore a prediction of the tensile strength is difficult [33].

Since this theory is based on saturation, the BL needs to be transferred following Equation (9):

(9) $$S\;=\;\frac{\mathrm{BL}}{100}*\frac{\rho_{\mathrm{bulk}}}{\rho_{\mathrm{ink}}\cdot \left(1\;-\;\frac{\rho_{\mathrm{bulk}}}{\rho_{\mathrm{solid}}}\right)}$$

with S as the saturation, BL as the Binder Loading, ρ_bulk_ as the bulk density, ρ_ink_ as the density of the solvent, and ρ_solid_ as the material density of the particle.

For the printed parts up to a BL of 15% (corresponding to a saturation of 27.98%), we assume bridge dominated behavior. Figure 7 provides more evidence for this assumption. In the images up to a BL of 15%, the particles are linked by bridges (Figure 7a–c). At 20 wt% (Figure 7d), the particulate structure is less pronounced but still present. At higher binder loadings (Figure 7e–g), the particulate structure is not visible anymore. As the structure is very different, different explanations for the correlation between tensile strength and porosity are necessary. This correlates well with the wetting theory explained before.

**Figure 11:**
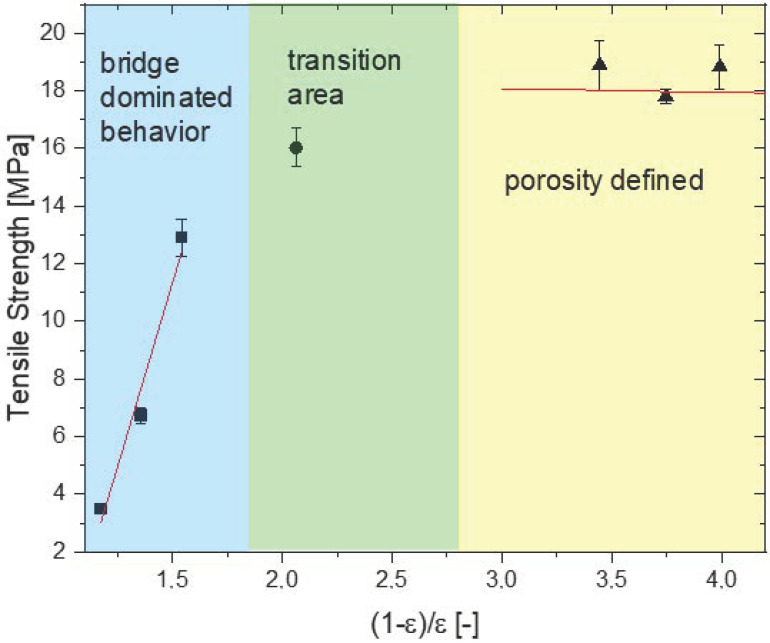
Tensile strength as a function of the quotient 1-ϵ and ϵ. Three regions can be identified: a linear correlation for BL up to 15% (bridge dominated behavior), a transition area, and porosity defined regime.

In order to use the model from Rumpf (Equation (9)) in Figure 11, the tensile strength is plotted against the ratio between 1 − ϵ and ϵ which is a measure for the solid particle volume in relationship to the porous volume (solids fraction). Three different areas can be distinguished.

The values of (1 − ϵ)/ϵ < 1.55 represent the binder loadings at 15 wt% and below. These values correspond to a bridge dominated behavior (see also Figure 7a–c), and therefore the Rumpf model can be applied. Using the slope of the graph and the particle size d_50,3_, the adhesion force is calculated to 0.077 N (see Figure 11, bridge dominated behavior).

Between 15% and 25% BL, a transition region exists where a part of the structure is dominated by bridges and a part by solid areas (Figure 7d). Thus, the tensile strength can be approximated by a linear interpolation between the two regions as follows:

(10) $$\sigma_{z}{\;=\;\sigma }_{z,\;S\;=\;0.28}\;+\;(S\;-\;0.28)\cdot \frac{\sigma_{z,S\;=\;0.58}\;{-\;\sigma }_{z,S\;=\;0.28}}{0.58\;-\;0.28}$$

S = 0.28 and S = 0.58 were calculated using Equation (9) and correspond to a BL of 15% and 25%, respectively. A graph of the tensile strength plotted against the saturation can be found in the Supplementary Information Figure S2.

A further increase in the BL to 25% suddenly changes the internal microstructure and no particulate bridges are visible (see Figure 7e–g), therefore the ratio (1 − ϵ)/ϵ changes from 2 to above 3.4. As discussed above, higher binder loadings do not change porosity, pore size distribution, or mechanical properties much. The variation in the solid fraction (1 − ϵ)/ϵ is due to the high sensitivity of the term (1 − ϵ)/ϵ at low porosities (see Figure 11, porosity defined). In this area, we propose that the porosity defines the tensile strength. The excess stress due to the pores when applying an external force and the reduced cross section lead to highly reduced values compared to cased pore free PMMA.

Some other empirical models exist for the relationship between porosity and tensile strength of ceramics [35,36]. Compared to these models, the proposed model has the advantage that it is developed from a bottom-up approach compared to an empirical fit afterwards. Further, Günther and Mögele [3] published a basic model to estimate the maximum tensile strength in binder jetting. Their basic model describes the material bridges between particles as cylinder minus two cones. The model from Günther and Mögele has some assumption which do not describe the reality in a powder bed, e.g., ideal spherical particles of the same size and cubic packaging. Therefor the knowledge from agglomeration theory developed by Rumpf seems to be a better fit for low binder loadings and bridge defined structures.

## 5. Conclusions

Within this study it has been shown that with a solvent bonding mechanism, the morphology, mechanical properties, and pore sizes of 3D printed parts can be controlled. With lower binder loadings, a high shape accuracy can be achieved, while higher loadings give better mechanical properties and smaller pore sizes. By setting the BL, the median pore size could be adjusted from 22 µm down to 6 µm. Within the investigated material, combination pore sizes of 6 µm were achieved, and their functionality was shown in a filter experiment. Optimal printing conditions are 20–30% BL, depending on the application. For smaller pores, higher loadings are required, and for a better shape accuracy, lower loadings are favorable. To demonstrate the feasibility of this approach, the results were tested in a filtration setup and could separate a particulate suspension very well. Further the obtained results were correlated to the works of Rumpf and Schubert, who studied the capillary forces within agglomerates and are based on a very similar mechanism for binding. Therefore, the equation of Rumpf could be applied to describe a relationship between porosity and tensile strength, and the adhesion force is calculated to 0.077 N.

The presented work is an important step to better understanding the powder binder interaction and its effect on macroscopic properties, such as tensile strength, but also on the microscopic structure. With the good scalability and high process speed of binder jetting technology, we hope this research is highly valuable for membrane manufacturers, pharma industry and catalyst manufacturers.

## Figures and Tables

**Table 1:** Print settings at different binder loadings.

Target Binder Loading (%)	5	10	15	20	25	30	45
Calculated binder loading	4.95	9.87	14.20	19.25	25.76	29.65	45.24
dx/dy (µm)	240/50	150/50	100/50	80/50	53/50	45/50	25/50
Print frequency (Hz)	1500	3000	4000	6000	6000	6000	8000
Droplet mass (ng)	67.87	89.16	90.14	103.90	98.76	103.13	111.33

## Data Availability

The data presented in this study are available on request from the corresponding author. The data are not publicly available due to industrial cooperation with Evonik Operations GmbH.

## Supplementary Materials

The following are available online at https://www.mdpi.com/1996-1944/14/5/1190/s1, Figure S1: Cross section of tensile road with surface area measurement, Figure S2: Tensile strength as a function of Saturation with linear interpolation in the transition area to predict the Tensile strength.

## References

[1] Sachs E.M., Haggerty J.S., Cima M.J., Williams P.A. (1994). Three-Dimensional Printing Techniques. US Patent.

[2] Gebhardt A. (2016). Additive Fertigungsverfahren.

[3] Günther D., Mögele F., Shishkovsky I.V. (2016). Additive Manufacturing of Casting Tools Using Powder-Binder-Jetting Technology. New Trends in 3D Printing.

[4] Mostafaei A., Elliott A.M., Barnes J.E., Li F., Tan W., Cramer C.L., Nandwana P., Chmielus M. (2020). Binder jet 3D printing—Process parameters, materials, properties, and challenges. Prog. Mater. Sci..

[5] He Z., Shanmugasundaram T.S., Singh G. (2018). Inkjet 3D printing of clay ceramics for water treatment. Prog. Addit. Manuf..

[6] Hwa L.C., Uday M.B., Ahmad N., Noor A.M., Rajoo S., Zakaria K.B. (2018). Integration and fabrication of the cheap ceramic membrane through 3D printing technology. Mater. Today Commun..

[7] Lee J.-Y., Tan W.S., An J., Chua C.K., Tang C.Y., Fane A.G., Chong T.H. (2016). The potential to enhance membrane module design with 3D printing technology. J. Membr. Sci..

[8] Low Z.-X., Chua Y.T., Ray B.M., Mattia D., Metcalfe I.S., Patterson D.A. (2017). Perspective on 3D printing of separation membranes and comparison to related unconventional fabrication techniques. J. Membr. Sci..

[9] Tijing L.D., Dizon J.R.C., Ibrahim I., Nisay A.R.N., Shon H.K., Advincula R.C. (2019). 3D printing for membrane separation, desalination and water treatment. Appl. Mater. Today.

[10] Goole J., Amighi K. (2016). 3D printing in pharmaceutics: A new tool for designing customized drug delivery systems. Int. J. Pharm..

[11] Brunello G., Sivolella S., Meneghello R., Ferroni L., Gardin C., Piattelli A., Zavan B., Bressan E. (2016). Powder-based 3D printing for bone tissue engineering. Biotechnol. Adv..

[12] Ke D., Bose S. (2018). Effects of pore distribution and chemistry on physical, mechanical, and biological properties of tricalcium phosphate scaffolds by binder-jet 3D printing. Addit. Manuf..

[13] Yu D.G., Yang X.L., Huang W.D., Liu J., Wang Y.G., Xu H. (2007). Tablets with material gradients fabricated by three-dimensional printing. J. Pharm. Sci..

[14] Spath S., Drescher P., Seitz H. (2015). Impact of particle size of ceramic granule blends on mechanical strength and porosity of 3D printed scaffolds. Materials.

[15] Polzin C., Günther D., Seitz H. (2015). 3D printing of porous Al_2_O_3_ and SiC ceramics. J. Ceram. Sci. Technol..

[16] Maleksaeedi S., Eng H., Wiria F.E., Ha T.M.H., He Z. (2014). Property enhancement of 3D-printed alumina ceramics using vacuum infiltration. J. Mater. Process. Technol..

[17] Zocca A., Gomes C.M., Staude A., Bernardo E., Günster J., Colombo P. (2013). SiOC ceramics with ordered porosity by 3D-printing of a preceramic polymer. J. Mater. Res..

[18] Rumpf H.C.H. (1970). Zur Theorie der Zugfestigkeit von Agglomeraten bei Kraftübertragung an Kontaktpunkten. Chem. Ing. Tech..

[19] Schubert H. (1975). Tensile strength of agglomerates. Powder Technol..

[20] Schubert H. (1979). Grundlagen des Agglomerierens. Chem. Ing. Tech..

[21] Ramakrishnan R. (2015). 3-D-Drucken Mit Einem Anorganischen Formstoffsystem. Ph.D. Thesis.

[22] DIN (2019). Plastics—Determination of Tensile Properties—Part 1: General Principles.

[23] ASTM International (2015). B962-17.

[24] Albrecht K., Stickler M., Rhein T. (2013). Polymethacrylates. Ullmann’s Encyclopedia of Industrial Chemistry.

[25] Jena A., Gupta K. (2001). An innovative technique for pore structure analysis of fuel cell and battery components using flow porometry. J. Power Sources.

[26] Kellner I. (2013). Materialsysteme für das Pulverbettbasierte 3D-Drucken.

[27] Schmutzler C., Stieh T.H., Michael F.Z. (2019). Empirical process model for shrinkage-induced warpage in 3D printing. Rapid Prototyp. J..

[28] Schmutzler C., Zeller C., Amann S., Seidel C., Zäh M.F. Simulation des Verzugs Infolge des Schichtweisen Aufbaus im 3-D-Druck. Proceedings of the ANSYS Conference and 33. CADFEM Users Meeting 2015.

[29] Pfaff F.A., Gelfant F.S. (1997). Osmotic blistering of epoxy coatings on concrete. J. Prot. Coat. Linings.

[30] Polzin C., Spath S., Seitz H. (2013). Characterization and evaluation of a PMMA-based 3D printing process. Rapid Prototyp. J..

[31] Patirupanusara P., Suwanpreuk W., Rubkumintara T., Suwanprateeb J. (2008). Effect of binder content on the material properties of polymethyl methacrylate fabricated by three dimensional printing technique. J. Mater. Process. Technol..

[32] Schubert H. (1973). Kapillardruck und Zugfestigkeit von feuchten Haufwerken aus körnigen Stoffen. Chem. Ing. Tech..

[33] Stieß M. (1997). Mechanische Verfahrenstechnik 2.

[34] Stieß M. (2009). Mechanische Verfahrenstechnik. Partikeltechnologie 1.

[35] Melcher R.R. (2009). Rapid Prototyping von Keramiken Durch 3D-Drucken.

[36] Chen X., Wu S., Zhou J. (2013). Influence of porosity on compressive and tensile strength of cement mortar. Constr. Build. Mater..

