# Supplementary material for: The Effect of Binder Loading on the Pore Size of 3D Printed PMMA
Source: Materials (Basel). 2021 Mar 3;14(5):1190. doi: 10.3390/ma14051190 (PMC7959472; doi:10.3390/ma14051190)
Supplement: Supplementary file 1 [file materials-14-01190-s001.pdf]

materials

MDPI

Supplementary Material

The Effect of Binder Loading on the Pore Size of 3D Printed PMMA

Simon Riechmann [1,2,*], Odo Wunnicke [2] and Arno Kwade [1]

[1] Institut für Partikeltechnik, TU Braunschweig, Volkmaroder Str. 5, 38104 Braunschweig, Germany; a.kwade@tu-braunschweig.de
[2] Evonik Operations GmbH, Creavis, Paul-Baumann-Str. 1, 45764 Marl, Germany; odo.wunnicke@evonik.com
* Correspondence: simon.riechmann@tu-braunschweig.de

Citation: Riechmann, S.; Wunnicke, O.; Kwade, A. The Effect of Binder Loading on the Pore Size of 3D Printed PMMA. *Materials* **2021**, *14*, 1190. https://doi.org/10.3390/ma14051190

Academic Editor: Massimiliano Avalle

Received: 31 January 2021
Accepted: 23 February 2021
Published: 3 March 2021

Publisher's Note: MDPI stays neutral with regard to jurisdictional claims in published maps and institutional affiliations.

Copyright: © 2021 by the authors. Submitted for possible open access publication under the terms and conditions of the Creative Commons Attribution (CC BY) license (http://creativecommons.org/licenses/by/4.0/).

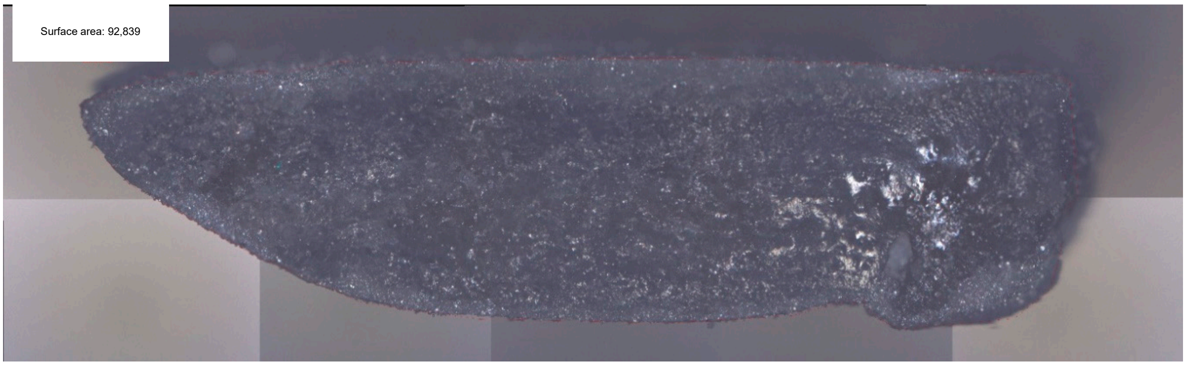

Figure S1. Cross sect ion of tensile road with surface area measurement. This is used to adjust the Tensile strength at 45% BL. Originally the width of 20.04 mm and height 5.28 mm were measured with a caliper.

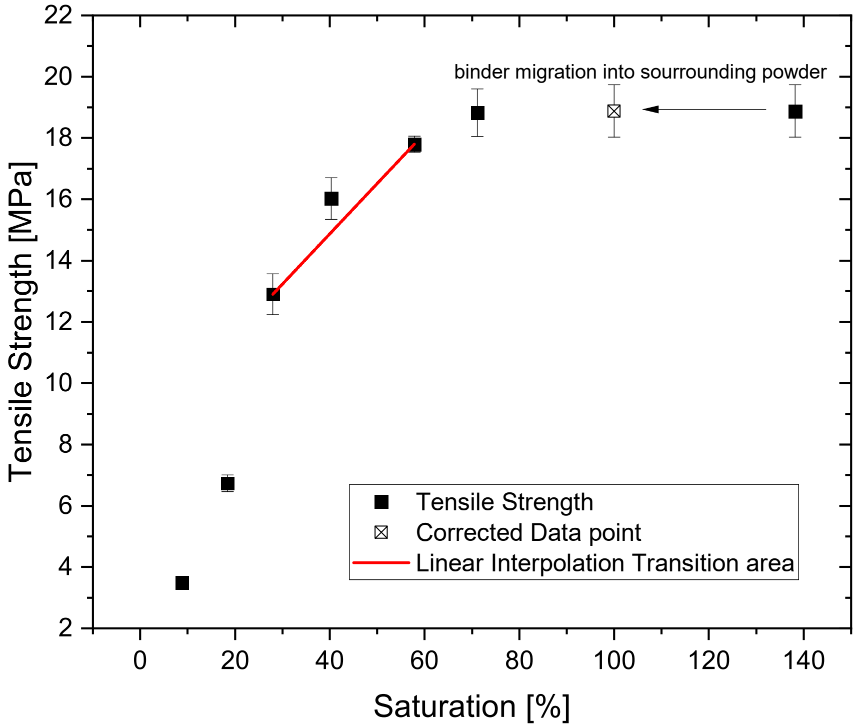

Figure S2. Tensile strength as a function of Saturation with linear interpolation in the transition area to predict the Tensile strength. The print job with 45% BL calculates to a Saturation over 100%. This is not possible and explains the high shape deviation. Due to the binder migration into surrounding powder it is assumed that a BL of 100% is achieved.
